# c-*erb*B-4 protein expression in human breast cancer

**DOI:** 10.1054/bjoc.1999.1057

**Published:** 2000-02-21

**Authors:** T Y Kew, J A Bell, S E Pinder, H Denley, R Srinivasan, W J Gullick, R I Nicholson, R W Blamey, I O Ellis

**Affiliations:** The Breast Unit, Nottingham City Hospital, Hucknall Road, Nottingham, NGS IPB, UK; Receptor Biology Laboratory, Imperial Cancer Research Fund Molecular Oncology Unit, Imperial College School of Medicine, Hammersmith Hospital, London, UK; Tenovus Institute, Cardiff, UK

**Keywords:** breast cancer, c-*erb*B-4, growth factor receptor, prognostic factors

## Abstract

The Type 1 family of growth factor receptors includes epidermal growth factor receptor (EGFR), c- *erb* B-2, c- *erb* B-3 and c- *erb* B-4. Overexpression of the first two members is associated with poorer prognosis in patients with breast carcinoma. In this study we examined the expression of c- *erb* B-4 protein using the monoclonal antibody HFR-1. A total of 127 consecutive cases of primary operable invasive breast carcinoma presenting between 1975 and 1977 were studied. All patients were managed by simple mastectomy or conservation surgery with radiotherapy and no adjuvant therapy given. Long-term follow-up was maintained. Routine, formalin-fixed, paraffin-embedded tumour samples were used and sections were stained immunohistochemically using the Duet StreptABC method. Immunoreactivity was classified using a simple semi-quantitative scoring method. Protein expression was generally low but definite positive cytoplasmic, membranous and nuclear reactivity was identified in 58%, 41% and 25% of cases respectively. Expression at all three sites demonstrated significant inverse associations were histological grade. In addition, membrane accentuation correlated inversely with the Nottingham Prognostic Index (NPI), while cytoplasmic reactivity showed a positive association with c- *erb* B-3 expression. No significant associations were found with disease-free interval or survival. The results of this study demonstrate that higher levels of c- *erb* B-4 protein expression are associated with a more differentiated histological phenotype in contrast to the other members of the Type 1 family. Larger series with extended follow-up will be required to ascertain definitively the prognostic value of c- *erb* B-4 expression in breast carcinoma. © 2000 Cancer Research Campaign


*British Journal of Cancer* (2000) **82**(6), 1163–1170
© 2000 Cancer Research Campaign
Article no. bjoc.1999.1057, available online at http://www.idealibrary.com on IDEAL®

### c-*erb*B-4 protein expression in human breast cancer

**TY Kew[1], JA Bell[1], SE Pinder[1], H Denley[1], R Srinivasan[2], WJ Gullick[2], RI Nicholson[3], RW Blamey[1] and IO Ellis[1]**

[1]The Breast Unit, Nottingham City Hospital, Hucknall Road, Nottingham NGS IPB, UK; [2]Receptor Biology Laboratory, Imperial Cancer Research Fund Molecular Oncology Unit, Imperial College School of Medicine, Hammersmith Hospital, London, UK; [3]Tenovus Institute, Cardiff, UK

**Summary** The Type 1 family of growth factor receptors includes epidermal growth factor receptor (EGFR), c-*erb*B-2, c-*erb*B-3 and c-*erb*B-4. Overexpression of the first two members is associated with poorer prognosis in patients with breast carcinoma. In this study we examined the expression of c-*erb*B-4 protein using the monoclonal antibody HFR-1. A total of 127 consecutive cases of primary operable invasive breast carcinoma presenting between 1975 and 1977 were studied. All patients were managed by simple mastectomy or conservation surgery with radiotherapy and no adjuvant therapy given. Long-term follow-up was maintained. Routine, formalin-fixed, paraffin-embedded tumour samples were used and sections were stained immunohistochemically using the Duet StreptABC method. Immunoreactivity was classified using a simple semi-quantitative scoring method. Protein expression was generally low but definite positive cytoplasmic, membranous and nuclear reactivity was identified in 58%, 41% and 25% of cases respectively. Expression at all three sites demonstrated significant inverse associations were histological grade. In addition, membrane accentuation correlated inversely with the Nottingham Prognostic Index (NPI), while cytoplasmic reactivity showed a positive association with c-*erb*B-3 expression. No significant associations were found with disease-free interval or survival. The results of this study demonstrate that higher levels of c-*erb*B-4 protein expression are associated with a more differentiated histological phenotype in contrast to the other members of the Type 1 family. Larger series with extended follow-up will be required to ascertain definitively the prognostic value of c-*erb*B-4 expression in breast carcinoma. © 2000 Cancer Research Campaign

**Keywords:** breast cancer; c-*erb*B-4; growth factor receptor; prognostic factors

Four distinct transmembranous glycoprotein receptors have been identified as members of the Type 1 growth factor receptor family, a class of oncogenes prevalent in several solid tumours, especially those of the breast (reviewed in Mason and Gullick, 1995). They are the epidermal growth factor receptor (EGFR), c-*erb*B-2, c-*erb*B-3 and c-*erb*B-4 (alternatively, authors may use the HER terminology instead, e.g. HER1 etc). Receptor heterodimerization (Earp et al, 1995), a unique feature of the Type 1 receptors, enables multifunctional intracellular responses to be generated with differing strengths and specificities dependent on the ligand type, specific receptor combination or relative level of receptor expression. Previous studies have shown that overexpression of EGFR, c-*erb*B-2 and c-*erb*B-3 is associated with worse disease prognosis in breast cancer (Slamon et al, 1987; Lewis et al, 1990, 1996; Lovekin et al, 1991; Klijn et al, 1992; Lemoine et al, 1992; Travis et al, 1996). The most common causes of Type 1 receptor overexpression are gene amplification and/or an increased gene transcription rate. Much interest has been generated recently by the possibility of utilizing the Type 1 receptors as prognostic indicators in breast carcinomas and the development of treatments in the form of kinase inhibitors and monoclonal antibodies (Bridges, 1996).

*Received 22 March 1999*
*Revised 16 September 1999*
*Accepted 20 September 1999*

*Correspondence to:* IO Ellis, Department of Histopathology, Nottingham City Hospital, Hucknall Road, Nottingham NG5 1PB, UK.
E-mail: ian.ellis@nottingham.ac.uk

The most recently identified member of this family, c-*erb*B-4, was cloned in 1993 (Plowman et al, 1993). The c-*erb*B-4 gene is localized on chromosome 2q33.3–34 (Zimonjic et al, 1995) and encodes a protein of 1283 amino acids with a molecular weight of 180 kDa after post-translational glycosylation. Although EGFR, c-*erb*B-2 and c-*erb*B-3 are all expressed as soluble, truncated receptors as a result of mRNA splicing, c-*erb*B-4 exists as two full-length variants possessing alternative extracellular juxtamembrane sequences which are differentially sensitive to proteolysis (Elenius et al, 1997*b*). Protein kinase C activators (e.g. 12-*O*-tetradecanoylphorbol-13-acetate, platelet-derived growth factor) have been shown to induce rapid and extensive selective proteolytic cleavage of the c-*erb*B-4 receptor molecule, yielding a membrane-dependent 80-kDa fragment consisting of the entire cytoplasmic and transmembrane domain, and a soluble 120-kDa ectodomain fragment which is released into the extracellular space (Vecchi et al, 1996; Vecchi and Carpenter, 1997).

Expression of the c-*erb*B-4 receptor is found in several breast adenocarcinoma cell lines (e.g. MDA-MB-453, T47-D, BT-474 and H3396), and in a range of normal human fetal and adult tissues at the mRNA (Plowman et al, 1993*a*); Srinivasan et al, 1998) and protein levels (Srinivasan et al, 1998), notably in the brain and heart. More specifically, c-*erb*B-4 has also been found expressed at the post-synaptic membrane of neuromuscular synapses (Zhu et al, 1995). The c-*erb*B-4 receptor is activated by ligands known as heregulins or neuregulins (Plowman et al, 1993*b*). These are encoded by three separate genes, NRG-1 (the human homologue of the mouse NDF gene) found at chromosome 8p12–21 (Orr-Urteger et al, 1993), and two more recently described genes NRG-2 (also known as Don1 and NTAK) (Busfield et al, 1997; Carraway et al, 1997; Chang et al, 1997; Higashiyama et al, 1997)

and NRG-3 located on chromosome 10q22 (Zhang et al, 1997). The first two of these genes are known to produce multiple proteins possessing different structural domains as a consequence of mRNA splicing (Wen et al, 1994). In addition, several ligands originally described as activators of EGFR are now known also to bind directly to and to stimulate the activity of c-*erb*B-4. Examples of these include betacellulin (Riese et al, 1996), heparin-binding EGF (Elenius et al, 1997*a*) and epiregulin (Komurasaki et al, 1997).

Consistent with its pattern of expression, knock-out mice studies have suggested that c-*erb*B-4 plays an essential role as an in vivo regulator of both cardiac muscle differentiation and axonal guidance in the central nervous system (Gassmann et al, 1995; Zhu et al, 1995). Gassman et al reported that mice lacking c-*erb*B-4 died during mid-embryogenesis from the aborted development of myocardial trabeculae in the heart ventricle. In addition, alterations of the innervation of the hindbrain were also reported.

The relationship between c-*erb*B-4 expression and cancer prognosis is, at present, poorly understood. Srinivasan et al (1998) found that the immunohistochemical expression of c-*erb*B-4 tended to be higher in normal tissues than in most of the types of cancers studied. Similar results have been obtained by Lyne et al (1997) in prostate cancer and by Baccus et al (1996) breast carcinoma, the latter also demstrating divergent correlates between c-*erb*B-2 and c-*erb*B-4 (Baccus et al, 1996). In this study, we demonstrated the immunohistochemical expression of c-*erb*B-4 in breast cancer using the HFR-1 monoclonal antibody. We also examined for possible correlations between its expression and various indicators of clinical prognosis, with a view to assessing the possible value of c-*erb*B-4 expression as an independent prognostic indicator in breast carcinomas.

### MATERIALS AND METHODS

### Patients

A consecutive series of 133 patients presenting with primary operable invasive breast carcinoma to a single surgeon (RW Blamey) at the Nottingham City Hospital between 1975 and 1977 was examined. The treatment administered involved mastectomy (simple or subcutaneous) or breast conservation surgery with radiotherapy. During surgery itself, lymph nodes were sampled by the triple node biopsy technique (Todd et al, 1987) and the tumour subsequently staged. No systemic adjuvant therapy was administered. Long-term follow-up of patients was maintained after surgery by regular visits to the clinic. These were conducted at 3-monthly intervals for 18 months, and then every 6 months for 5 years, and then annually. Any major tumour recurrence was recorded as either loco-regional (recurrences requiring some form of major treatment, e.g. radiotherapy) or distant (confirmed radiologically by isotope scan or liver function tests). The disease-free interval was taken as the time (in months) from the date of primary treatment to the first loco-regional or distant recurrence. The overall survival was defined as the time (in months) from the date of primary treatment to the time of death.

Of the 133 patients entered into the study, six were removed because data on certain prognostic variables was not available from the computer database. This leaves a total of 127 patients. All patients were aged 70 years or below.

### Tissue preparation

The excised tumours were sliced and measured in three perpendicular planes. This was done immediately post-excision to reduce the possibility of autolytic artifacts. The largest of these three dimensions constituted the actual tumour size recorded. Fresh tumour blocks were snap-frozen or fixed in neutral buffered formalin and then embedded in paraffin wax for receptor assay, immunochemistry and archival storage. Histological grade (Elston and Ellis, 1991), tumour type (Ellis et al, 1992), menopausal status (Todd et al, 1987) and vascular invasion (Pinder et al, 1994) were recorded for each tumour sample. A Nottingham Prognostic Index (NPI) score was calculated for each patient based on the following equation (Haybittle et al, 1982):

$$I = 0.2 \times \text{size (in cm)} + \text{stage} + \text{grade}$$

Patients were then assigned into the good ($I \leq 3.4$), moderate ($3.4 < I \leq 5.4$) or poor ($I > 5.4$) prognostic groups according to the score obtained. In addition, assays to determine the oestrogen receptor (ER) status of each tumour section were conducted (at the Tenovus Institute, Cardiff) by the dextran-coated charcoal method. Tumours were classified ER-positive if they contained more than 5 femtomoles of specific oestradiol binding per mg of cytosol protein (Todd et al, 1987). Finally, tumour sections were divided into four different histological groups with different prognostic indications (Pereira et al, 1995):

Group 1: Tubular, tubulo-lobular, mucoid and invasive cribriform carcinoma.
Group 2: Tubular mixed, mixed ductal with special type, and alveolar lobular carcinoma.
Group 3: Classical lobular, medullary, atypical medullary and lobular mixed.
Group 4: Ductal NST, solid lobular, mixed ductal and lobular carcinoma.

### Immunohistochemistry

The tumour samples were then stained immunohistochemically with the HFR-1 monoclonal antibody using the Duet StreptABC Method (A = streptavidin, B = biotinylated peroxidase, C = biotinylated goat anti-mouse/rabbit immunoglobulin) with DAB as the chromogen and haematoxylin as the counterstain. HFR-1 is a mouse monoclonal antibody raised against residues 1249–1264 of the c-*erb*B-4 cytoplasmic domain. It has been shown to recognize c-*erb*B-4 by immunoprecipitation, Western blotting and immunostaining of cytocentrifuge preparations of NIH3T3 cells transfected with c-*erb*B-4 (Srinivasan et al, 1998). Initially, the antibody was supplied at a concentration of 1.5 mg $ml^{-1}$. After repeated trials to find a suitable dilution concentration with the most appropriate balance of protein expression and background staining, a ratio of 1/700 was decided upon, giving a final antibody concentration of approximately 2.14 μg $ml^{-1}$. Sections of normal breast tissue were used as positive controls, and omission of the primary antibody and blockade of the primary antibody with peptide 96.4 were used as negative controls.

*© 2000 Cancer Research Campaign*

The final protein expression was classified using a simple semi-quantitative scoring method, based on three distinct sites of reactivity. To increase the objectivity and accuracy of the scoring process, the shade of brown reaction product expressed in the cytoplasm was compared to a colour chart available commercially as the ICI Dulux Colour Palette™. The scoring criteria used are as follows:

1. Nuclear reactivity
   0 = No reactivity
   1 = Pale brown and finely granular
   2 = Dark opaque brown
2. Cytoplasmic reactivity
   0 = Clear cytoplasm
   1 = First shade of colour chart
   2 = Second shade of colour chart
   3 = Third shade of colour chart
3. Membrane accentuation
   0 = No membrane accentuation
   1 = 0–10% of cells demonstrating membrane accentuation
   2 = >10% of cells demonstrating membrane accentuation.

To ensure consistency of scoring, pairs of sections with similar scores were randomly chosen for re-assessment. The observed reactivities were compared between the two sections, and scores were re-adjusted if necessary. Approximately 20 sections were scored blind by two pathologists (IOE and HD) using the same scoring criteria. Results from Spearman rank correlation analysis indicated that the inter-observer agreement was good for cytoplasmic (Rho = 0.647) and membrane (Rho = 0.510) reactivity.

### Statistical analysis

The semi-quantitative scores assigned to each patient case were entered into a statistical software program which contained information on a variety of factors recorded routinely (see Table 1). This is the fourth in a series of similarly designed studies investigating the individual expression of Type 1 receptors in breast carcinoma patients, enabling us to utilize data on the expression of EGFR (Lewis et al, 1990), c-*erb*B-2 (Lovekin et al, 1991) and c-*erb*B-3 (Travis et al, 1996) for statistical analyses. Univariate $\chi^2$ analyses were conducted for each of the sites of reactivity mentioned and several known prognostic factors in breast cancer. Survival data were examined using the Kaplan–Meier method and the log-rank (Mantel–Cox) test. All analyses were carried out using standard commercial statistical computer software (Stat View 4.1).

### RESULTS

In this study, c-*erb*B-4 immunoreactivity using HFR-1 antibody was localized to ductal and lobular units (both normal and malignant) and accompanied by an acceptable level of background staining. Within malignant cells, immunoreactivity was predominantly cytoplasmic, although nuclear and membrane reactivity were also present. The level of c-*erb*B-4 protein expression in tumour cell populations was generally low (as such the scoring process more difficult than initially envisaged), but definite cytoplasmic, membrane and nuclear reactivity (score 2 or above) was identified in 58%, 41% and 25% of cases respectively. Although statistically significant correlations were demonstrated between these three sites of expression, careful analysis of the contingency tables revealed no definite pattern of interaction.

### Chi-square tests

Statistically significant associations were demonstrated between histological grade and c-*erb*B-4 nuclear (Table 1 and 4; Figure 4), cytoplasmic (Tables 2 and 5; Figure 5) and membrane expression (Tables 3 and 6; Figure 6). In contrast to the other Type 1

**Table 1** Results of chi square tests for c-erbB-4 nuclear reactivity

| Prognostic factor | Cut-off points | $\chi^2$ | $\chi^2$ *P*-value |
|---|---|---|---|
| Grade | 1, 2, or 3 | 10.781 | **0.0291**[a] |
| Lymph node stage | 1, 2, or 3 | 5.888 | 0.2077 |
| Local recurrence | Absent or present | 0.048 | 0.9761 |
| Distant metastases | Absent or present | 0.245 | 0.8845 |
| Vascular invasion | None or definite | 0.135 | 0.9346 |
| Tumour size | ≤ 1.5 or > 1.5 cm | 1.174 | 0.5559 |
| NPI groups | Good, moderate, or poor prognostic group | 3.112 | 0.5393 |
| Histological type | 1, 2, 3, 4 or 5 | 6.814 | 0.3384 |
| Age | < 40, 40–49, 50–59, 60–69 or > 70 years | 5.919 | 0.6563 |
| Menopausal status | Pre- or post-menopausal | 2.324 | 0.3128 |
| ER status | Negative or positive | 2.486 | 0.2886 |
| EGFR | Negative, mild, moderate or strong immunoreactivity[b] | 6.944 | 0.3260 |
| C-erbB-2 | Negative or positive[b] | 1.681 | 0.4314 |
| C-erbB-3 | Negative, mild, moderate or strong immunoreactivity[b] | 12.078 | 0.0602 |

See under Materials and Methods for detailed description of criteria for cut-off points. [a]Figures highlighted indicate statistically significant *P*-values ($P < 0.05$). [b]For further information on EGFR, c-*erb*B-2 and c-*erb*B-3 scoring criteria, refer to Lewis et al (1990), Lovekin et al (1991) and Travis et al (1996) respectively.

© 2000 Cancer Research Campaign

**Table 2** Results of chi square tests for c-erbB-4 cytoplasmic reactivity

| Prognostic factor | $\chi^2$ | $\chi^2$ ***P*-value** |
|---|---|---|
| Grade | 13.141 | **0.0408** |
| Lymph node stage | 13.554 | **0.0350** |
| Local recurrence | 0.566 | 0.9042 |
| Distant metastases | 0.662 | 0.8822 |
| Vascular invasion | 1.195 | 0.7541 |
| Tumour size | 4.767 | 0.1897 |
| NPI groups | 6.567 | 0.3628 |
| Histological type | 9.456 | 0.3963 |
| Age | 16.303 | 0.1778 |
| Menopausal status | 1.906 | 0.5922 |
| ER status | 1.132 | 0.7694 |
| EGFR | 15.012 | 0.0906 |
| C-*erb*B-2 | 2.514 | 0.4727 |
| C-*erb*B-3 | 23.913 | **0.0044** |

**Table 5** C-*erb*B-4 cytoplasmic reactivity vs grade (*P* = 0.0408)

| | 1 | (%) | 2 | (%) | 3 | (%) | Row total |
|---|---|---|---|---|---|---|---|
| 0 | 0 | (0) | 4 | (8) | 7 | (11) | 11 |
| 1 | 1 | (8) | 22 | (43) | 19 | (30) | 42 |
| 2 | 9 | (69) | 16 | (31) | 32 | (51) | 57 |
| 3 | 3 | (23) | 9 | (18) | 5 | (8) | 17 |
| Column total | 13 | (100) | 51 | (100) | 63 | (100) | 127 |

**Table 6** C-erbB-4 membrane accentuation vs grade (*P* = 0.0415)

| | 1 | (%) | 2 | (%) | 3 | (%) | Row total |
|---|---|---|---|---|---|---|---|
| 0 | 5 | (38) | 12 | (24) | 27 | (43) | 44 |
| 1 | 0 | (0) | 15 | (29) | 16 | (25) | 31 |
| 2 | 8 | (62) | 24 | (47) | 20 | (32) | 52 |
| Column total | 13 | (100) | 51 | (100) | 63 | (100) | 127 |

**Table 3** Results for c-*erb*B-4 membrane accentuation

| Prognostic factor | $\chi^2$ | $\chi^2$ ***P*-value** |
|---|---|---|
| Grade | 9.935 | **0.0415** |
| Lymph nodes stage | 3.016 | 0.5552 |
| Local recurrence | 3.500 | 0.1738 |
| Distant metastases | 0.094 | 0.9543 |
| Vascular invasion | 0.785 | 0.6753 |
| Tumour size | 1.999 | 0.3681 |
| NPI groups | 10.460 | **0.0334** |
| Histological type | 6.345 | 0.3857 |
| Age | 8.900 | 0.3508 |
| Menopausal status | 2.166 | 0.3385 |
| ER status | 1.287 | 0.5255 |
| EGFR | 8.717 | 0.1901 |
| C-*erb*B-2 | 0.761 | 0.6835 |
| C-*erb*B-3 | 7.012 | 0.3197 |

**Table 4** C-*erb*B-4 nuclear reactivity vs grade (*P* = 0.0291)

| | 1 | (%) | 2 | (%) | 3 | (%) | Row Total |
|---|---|---|---|---|---|---|---|
| 0 | 2 | (15) | 12 | (24) | 26 | (41) | 40 |
| 1 | 4 | (31) | 27 | (52) | 24 | (38) | 55 |
| 2 | 7 | (54) | 12 | (24) | 13 | (21) | 32 |
| Column total | 13 | (100) | 51 | (100) | 63 | (100) | 127 |

receptors (Lewis et al, 1990; Lovekin et al, 1991; Travis et al, 1996), the relationship is an inverse one, i.e. c-*erb*B-4 expression tended to favour well differentiated tumours. In addition, c-*erb*B-4 membrane accentuation demonstrated an inverse association with the NPI: fewer tumours expressed c-*erb*B-4 with worsening prognostic grouping (Tables 3 and 7; Figure 7). Statistically significant associations were also seen between c-*erb*B-4 cytoplasmic expression and both c-erbB-3 expression and tumour stage (Table 2), although no distinct trend was identifiable from contingency tables (not shown). No correlations were found between the three sites of immunoreactivity for c-*erb*B-4 and the prognostic factors and follow-up events viz. overall survival or disease-free interval (DFI), local recurrence, distant metastasis, vascular invasion, tumour size, histological type, age, menopausal status and ER status.

### DISCUSSION

This study has demonstrated that c-*erb*B-4 protein expression can be identified in both normal and malignant breast epithelium. Expression in adenocarcinoma is most frequently cytoplasmic, with nuclear and membrane localization seen in a small proportion of cases. Other studies on the immunohistochemical expression of c-*erb*B-4 in tumours of the prostate (Lyne et al, 1997), thyroid (Faksvag Haugen et al, 1996) and in medulloblastoma (Gilbertson et al, 1997) also demonstrated predominantly cytoplasmic reactivity. Srinivasan et al (1998) however showed occasional nuclear staining in paraffin-embedded and in frozen sections of some

**Table 7** C-*erb*B-4 membrane accentuation vs NPI (*P* = 0.0334)

| | Good prognostic group | (%) | Average prognostic group | (%) | Poor prognostic group | (%) | Row Total |
|---|---|---|---|---|---|---|---|
| 0 | 10 | (34) | 19 | (28) | 15 | (50) | 44 |
| 1 | 3 | (10) | 23 | (34) | 5 | (17) | 31 |
| 2 | 16 | (55) | 26 | (38) | 10 | (33) | 52 |
| Column total | 29 | (100) | 68 | (100) | 30 | (100) | 127 |

 *© 2000 Cancer Research Campaign*

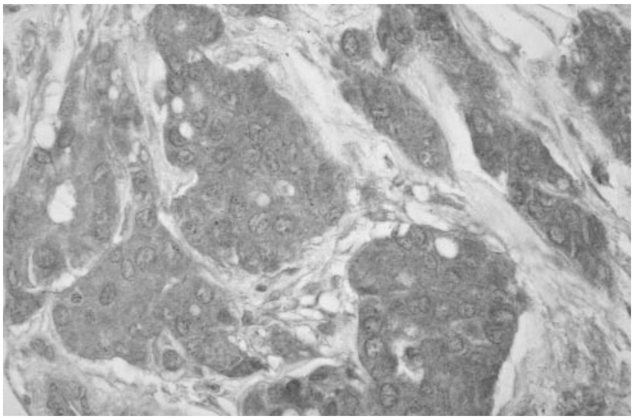

**Figure 1** C-*erb*B-4 cytoplasmic expression: the cytosol of this invasive carcinoma shows dense immunoreactivity (× 1600 original magnification)

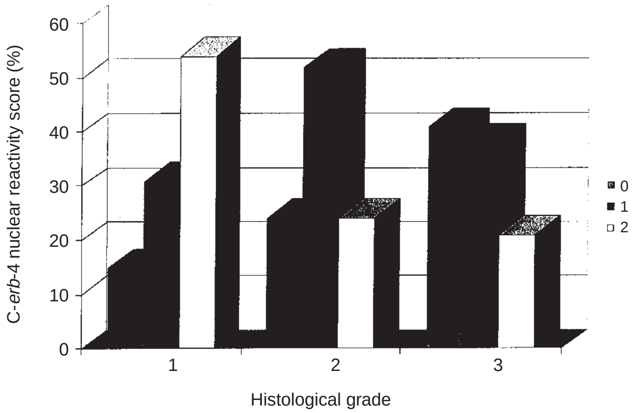

**Figure 4** With increasing histological grade, the percentage of tumour samples demonstrating score 0 (absent) nuclear reactivity increases, while that of tumours demonstrating score 2 (high) nuclear reactivity decreases

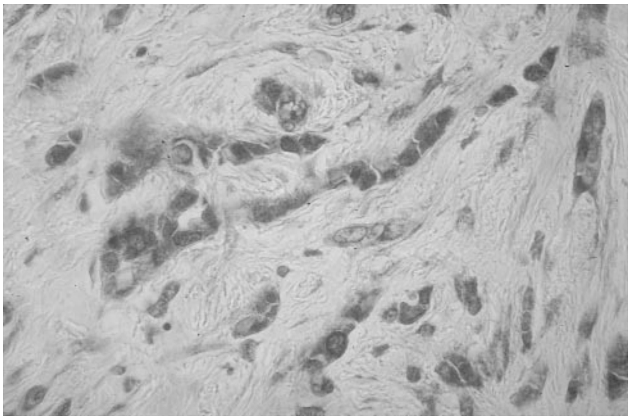

**Figure 2** C-*erb*B-4 nuclear expression: this invasive carcinoma shows some cytoplasmic reactivity but distinct nuclear reactivity is also visible (× 1600 original magnification)

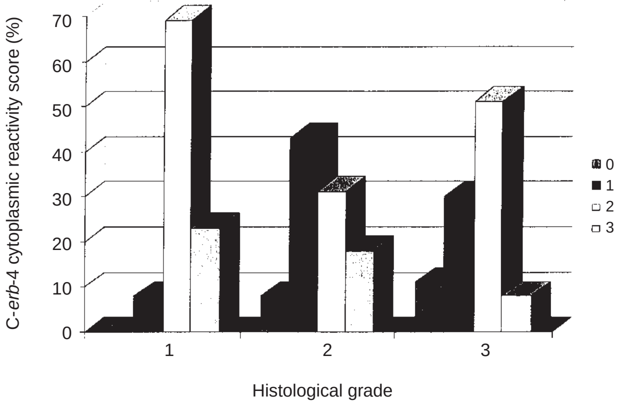

**Figure 5** Increasing percentage of tumour samples demonstrating score 0 cytoplasmic reactivity with increasing histological grade, and vice versa for score 3 cytoplasmic reactivity

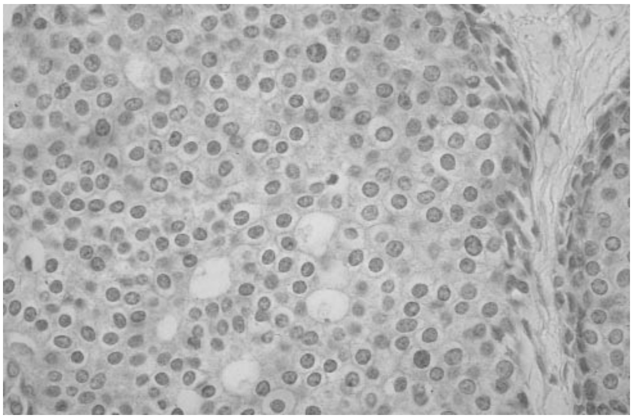

**Figure 3** C-*erb*B-4 membrane accentuation: the cellular membranes show light but distinct reactivity in this case of low grade cribriform DCIS (× 1600 original magnification)

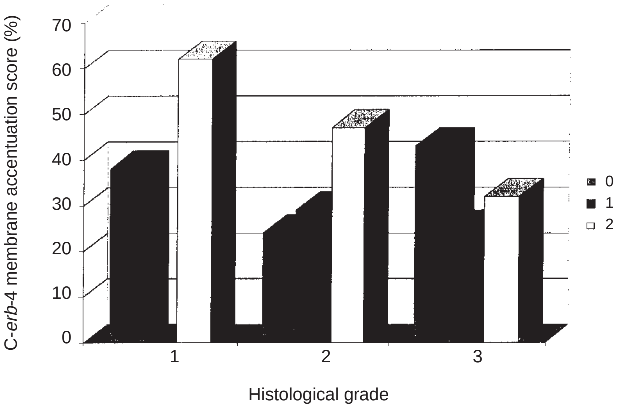

**Figure 6** Decreasing percentage of tumour samples demonstrating score 2 membrane accentuation with increasing histological grade

© 2000 Cancer Research Campaign

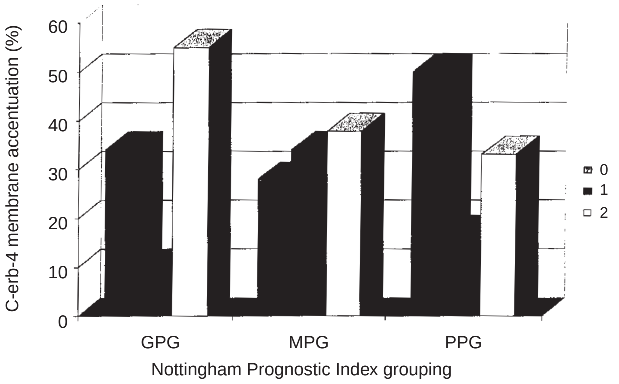

**Figure 7** Decreasing percentage of tumour samples demonstrating score 2 membrane accentuation with worsening prognostic grouping. NPI, Nottingham Prognostic Group; GPG, good prognostic group; MPG, moderate prognostic group; PPG, poor prognostic group normal tissues, including the breast. In this study, tumours with little or no expression of c-*erb*B-4 tended to possess characteristics associated with poorer prognosis in breast cancer i.e. histological grade 3 and the NPI poor prognosis grouping (PPG). These results suggest that an underlying mechanism of underexpression is present, which is a significantly divergent finding. Studies on the other members of the Type 1 family have shown repeatedly that overexpression of the receptor is correlated with poorer prognosis and shorter survival (Slamon et al, 1987; Lewis et al, 1990, 1996; Klijn et al, 1992; Lemoine et al, 1992).

Srinivasan et al (1998) described a similar pattern of receptor underexpression in their study on the expression of c-*erb*B-4 in nine common human malignancies using the same monoclonal antibody, in which they found less than normal expression of c-*erb*B-4 in 40–80% of these malignancies and in 100% of squamous cell carcinomas of the head and neck. Another study reported that loss of c-*erb*B-4 expression also occurs in prostatic cancers as compared to benign lesions and normal prostatic tissue (Lyne et al, 1997). However, a study on papillary thyroid carcinomas found overexpression of c-*erb*B-4 in 64% of cases (Faksvag Haugen et al, 1996). Knowlden et al (1998) examined c-*erb*B-3 and c-*erb*B-4 mRNA expression in human breast carcinoma and found increased expression of both to be associated with the prognostically favourable ER phenotype.

With regard to the other members of the Type 1 family, gene amplification and/or an increase in gene transcription have been shown to be responsible for protein overexpression in tumour samples (Kageyama et al, 1988; Hollywood and Hurst, 1993). The exact role of these receptors in the pathogenesis of breast cancer is still being elucidated, but we can speculate that overexpression of an oncogene encoding a putative growth factor receptor would give a growth advantage to the cells expressing it. In the case of c-*erb*B-4, however, underexpression of the receptor seems to be correlated with poorer clinical prognosis of breast cancer, possibly suggesting a mechanism akin to that of tumour suppressor genes, whereby normal suppression of mitogenesis is obliterated by loss of function mutations.

The nuclear and cytoplasmic expression of c-*erb*B-4 raises some intriguing issues, since members of the Type 1 family have long been shown to be transmembranous glycoprotein receptors. The cytoplasmic reactivity observed in this study may represent the presence of the c-*erb*B-4 glycoprotein in the cytoplasm due to reduced ligand-induced metabolic turnover and down-regulation of the receptor. While binding of EGF to the EGFR rapidly induces the clustering of ligand-receptor complexes in clathrin-coated pits, internalization of the complexes, and finally lysosomal degradation of both EGF and its receptor, the other Type 1 members, including c-*erb*B-4, are not subject to rapid internalization and down-regulation (Baulida et al, 1996). Putative internalization codes (e.g. sequences $^{996}$QQGFF and $^{973}$FYRAL) which have been discovered within the cytoplasmic domain of EGFR are not preserved in the cytoplasmic domain of c-*erb*B-4 (Chang et al, 1993). Experiments are underway to address this issue using GFP-tagged receptors and digital microscopy.

The nuclear expression, on the other hand, is something of an enigma, although it has been demonstrated that several of the neuregulin isoforms do contain putative nuclear targeting sequences near their amino termini (Holmes et al, 1992). NRG1-β has also been reported to be internalized and efficiently transported to the nucleus in breast cancer cells (Li et al, 1996). Further elucidation of the complex signal transduction cascades elicited by the Type 1 receptors may explain the nuclear expression of a receptor that at present has not been shown to be translocated to the nucleus.

The value of a c-erbB-4 immunohistochemical assay as a prognostic indicator in breast cancer is doubtful. As the spectrum of expression of c-*erb*B-4 in this study was shown to be relatively limited, semi-quantitative scoring would present difficulties with inter-observer variability. Although distinguishing between negative and positive expression may seem more easily reproducible, our analyses demonstrate that there is no significant correlation with patient prognosis even with this method (data not shown). Furthermore, c-*erb*B-4 expression demonstrated no significant association with either patient survival or disease-free interval.

In conclusion, the results of this study indicate that higher levels of c-*erb*B-4 protein expression are associated with a more differentiated histological phenotype. Immunoreactivity with the use of HFR-1 is generally low, although it does appear to have three separate components: cytoplasmic (which is predominant), nuclear and membranous. However, the immunoreactive expression of c-*erb*B-4 appears to be of limited prognostic value in breast carcinoma, although it may well prove to be of greater prognostic value in human malignancies other than the breast. Further studies may examine this possibility as well as the role of receptor heterodimerization in cancer progression and development. Indeed, a recent study on childhood medulloblastoma reported that the presence of c-*erb*B-4 expression with c-*erb*B-2 was indicative of tumour aggressiveness and poor prognosis (Gilbertson et al, 1997). We may discover that receptor heterodimerization among the Type 1 family of receptors plays a far more important role in human oncogenesis than individual receptors themselves (Earp et al, 1995). Further studies will be enhanced by our ability to measure all four Type 1 receptors in parallel with their known ligands and ligand variants.

 © 2000 Cancer Research Campaign

### REFERENCES

Bacus SS, Chin D, Yarden Y, Zelnick CR and Stern DF (1996) Type 1 receptor tyrosine kinases are differentially phosphorylated in mammary carcinoma and differentially associated with steroid receptors. *Am J Pathol* **148**: 549–558

Baulida J, Kraus MH, Alimandi M, Di Fiore PP and Carpenter G (1996) All ErbB receptors other than the epidermal growth factor receptor are endocytosis impaired. *J Biol Chem* **271**: 5251–5257

Bridges AJ (1996) The epidermal growth factor receptor family of tyrosine kinases and cancer: can an atypical exemplar be a sound therapeutic target? *Curr Med Chem* **3**: 167–194

Busfield SJ, Michnick DA, Chickering TW, Revett TL, Ma J, Woolf EA, Comrack CA, Dussault BJ, Woolf J, Goodearl AD and Gearing DP (1997) Characterisation of a neuregulin-related gene, Don-1, that is highly expressed in restricted regions of the cerebellum and hippocampus. *Mol Cell Biol* **17**: 4007–4014

Carpenter G (1987) Receptors for epidermal growth factor and other polypeptide mitogens. *Ann Rev Biochem* **56**: 881–914

Carraway KL, Weber JL, Unger MJ, Ledesma J, Yu N, Gasmann M and Lai C (1997) Neuregulin-2, a new ligand of ErbB3/ErbB4-receptor tyrosine kinases. *Nature* **387**: 512–516

Chang C-P, Lazar CS, Walsh BJ, Komuro M, Collawn JF, Kuhn LA, Tainer JA, Trowbridge IS, Farguhar MG, Rosenfeld MG, Wiley HS and Gill GN (1993) Ligand-induced internalization of the epidermal growth factor receptor is mediated by multiple endocytic codes analogous to the tyrosine motif found in constitutively internalized receptors. *J Biol Chem* **268**: 19312–19320

Chang H, Riese DJ, Gilbert W, Stern DF and McMahan UJ (1997) Ligands for the ErbB family receptors encoded by a neuregulin-like gene. *Nature* **387**: 509–512

Earp HS, Dawson TL, Lix and Yu H (1995) Heterodimerisation and functional interaction between EGF receptor family members: a new signalling paradigm with implications for breast cancer research. *Breast Cancer Res Treat* **35**: 115–132

Elenius K, Paul S, Allison G, Sun J and Klagsbrun M (1997*a*) Activation of HER4 by heparin-binding EGF-like growth factor stimulates chemotaxis but not proliferation. *EMBO J* **16**: 1268–1278

Elenius K, Corfas G, Paul S, Choi CJ, Rio C, Plowman GD and Klagsbrun M (1997*b*) A novel juxtamembrane domain isoform of HER4/ErbB4. *J Biol Chem* **272**: 26761–26768

Ellis IO, Galea M, Broughton N, Locker A, Blamey RW and Elston CW (1992) Pathological prognostic factors in breast cancer. II. Histological type. Relationship with survival in a large study with long-term follow-up. *Histopathology* **20**: 479–489

Elston CW and Ellis IO (1991) Pathological prognostic factors in breast cancer. I. The value of histological grade in breast cancer: experience from a large study with long-term follow-up. *Histopathology* **19**: 403–410

Faksvag Haugen DR, Akslen LA, Varhaug JE and Lillehaug JR (1996) Expression of c-erbB-3 and c-erbB-4 proteins in papillary thyroid carcinomas. *Cancer Res* **56**: 1184–1188

Gassmann M, Casagranda F, Orioli D, Simon H, Lai C, Klein R and Lemke G (1995) Aberrant neural and cardiac development in mice lacking the ErbB4 neuregulin receptor. *Nature* **378**: 390–394

Gilbertson RJ, Perry RH, Kelly PJ, Pearson ADJ and Lunec J (1997) Prognostic significance of HER2 and HER4 coexpression in childhood medulloblastoma. *Cancer Res* **57**: 3272–3280

Haybittle JL, Blamey RW, Elston CW, Johnson J, Doyle PJ, Campbell FC, Nicholson RI and Griffiths K (1982) A prognostic index in primay breast cancer. *Br J Cancer* **45**: 361–366

Higashiyama S, Horikawa M, Yamada K, Ichino N, Nakano N, Nakagawa T, Miyagawa J, Matsushita N, Nagatsu T, Taniguchi N and Ishiguro H (1997) A novel brain derived member of the epidermal growth factor family that interacts with ErbB3 and ErbB4. *J Biochem* **122**: 675–680

Hollywood DP and Hurst HC (1993) A novel transcription factor, OB2-1, is required for overexpression of the proto-oncogene c-*erb*B-2 in mammary tumour lines. *EMBO J* **12**: 2369–2375

Holmes WE, Sliwkowski MX, Akita RW, Henzel WJ, Lee J, Park JW, Yansura D, Abadi N, Raab H, Lewis GD, Shepard HM, Kuang W-J, Wood WI, Goeddel DV and Vandlen RL (1992) Identification of heregulin, a specific activator of p185$^{erb}$B2. *Science* **256**: 1205–1210

Kageyama R, Merlino T and Pastan I (1988) Epidermal growth factor (EGF) receptor gene transcription. Requirement for Sp1 and an EGF receptor-specific factor. *J Biol Chem* **263**: 6329–6336

Klijn JGM, Berns PMJJ, Schmitz PIM and Foekens JA (1992) The clinical significance of epidermal growth factor receptor (EGF-R) in human breast cancer: a review on 5232 patients. *Endocrine Rev* **13**: 3–17

Komurasaki T, Toyoda H, Uchida D and Morimoto S (1997) Epiregulin binds to epidermal growth factor receptor and ErbB-4 and induces tyrosine phosphorylation of epidermal growth factor receptor, ErbB-2, ErbB-3 and ErbB-4. *Oncogene* **15**: 2841–2848

Knowlden JM, Gee JMW, Seery LP, Farrow L, Gullick WJ, Ellis IO, Blamey RWB, Robertson JFR and Nicholson RI (1998) c-erbB3 and c-erbB4 expression is a feature of the endocrine responsive phenotype in clinical breast cancer. *Oncogene* **17**: 1949–1957

Lemoine NR, Barnes DM, Hollywood DP, Hughes CM, Smith P, Dublin E, Prigent SA, Gullick WJ and Hurst HC (1992) Expression of the ERBB3 gene-product in breast cancer. *Br J Cancer* **66**: 1116–1121

Lewis S, Locker A, Todd JH, Bell JA, Nicholson R, Elston CW, Blamey RW and IO Ellis (1990) Expression of epidermal growth factor receptor in breast carcinoma. *Clin Pathol* **43**: 385–389

Lewis GD, Lofgren JA, McMurtrey AE, Nuijens A, Fendly BM, Bauer KD and Sliwkowski MX (1996) Growth regulation of human breast and ovarian tumor cells by heregulin: evidence for the requirement of ErbB2 as a critical component in mediating heregulin responsiveness. *Cancer Res* **56**: 1457–1465

Li W, Park JW, Nuijens A, Sliwkowski MX and Keller GA (1996) Heregulin is rapidly translocated to the nucleus and its transport is correlated with c-*myc* induction in breast cancer cells. *Oncogene* **12**: 2473–2477

Lovekin C, Ellis IO, Locker A, Robertson JFR, Bell J, Nicholson R, Gullick WJ, Elston CW and Blamey RW (1991) C-erbB-2 oncoprotein expression in primary and advanced breast cancer. *Br J Cancer* **63**: 439–443

Lyne JC, Melham MF, Finley GG, Wen D, Liu N, Deng DH and Salup R (1997) Tissue expression of neu differentiation factor/heregulin and its receptor complex in prostate cancer and its biologic effects on prostate cancer cells in vitro. *Cancer J Scientific American* **3**: 21–30

Mason S and Gullick WJ (1995) Type 1 growth factor receptors: an overview of recent developments. *The Breast* **4**: 11–18

Pereira H, Pinder SE, Sibbering DM, Galea MH, Elston CW, Blamey RW, Robertson JFR and Ellis IO (1995) Pathological prognostic factors in breast cancer. IV: Should you be a typer or a grader? A comparative study of two histological prognostic features in operable breast carcinoma. *Histopathology* **27**: 219–226

Pinder SE, Ellis IO, Galea M, O'Rouke S, Blamey RW and Elston CW (1994) Pathological prognostic factors in breast cancer. III. Vascular invasion: relationship with recurrence and survival in a large study with long-term follow-up. *Histopath* **24(1)**: 41–47

Plowman GD, Culouscou J-M, Whitney GS, Green JM, Carlton GW, Foy L, Neubauer MG and Shoyab M (1993*a*) Ligand-specific activation of HER4/p180$^{erbB4}$, a fourth member of the epidermal growth factor receptor family. *Proc Natl Acad Sci USA* **90**: 1746–1750

Plowman GD, Green JM, Culouscou J-M, Carlton GW, Rothwell VM and Buckley S (1993*b*) Heregulin induces tyrosine phosphorylation of HER4/p180$^{erbB4}$. *Nature* **366**: 473–475

Riese DJ, Bermingham Y, Van Raaij TM, Buckley S, Plowman GD and Stern DF (1996) Betacellulin activates the epidermal growth factor receptor and erbB-4, and induces cellular response patterns distinct from those stimulated by epidermal growth factor or neuregulin-β. *Oncogene* **12**: 345–353

Slamon DJ, Clark GM, Wong SG, Levin WJ, Ullrich A and McGuire WL (1987) Human breast cancer: correlation of relapse and survival with amplification of the HER-2/*neu* oncogene. *Science* **235**: 177–182

Srinivasan R, Poulsom R, Hurst HC and Gullick WJ (1998) Expression of the c-erbB-4/HER4 protein and mRNA in normal human fetal and adult tissues and in a survey of nine solid tumour types. *J Path* **185**: 236–245

Todd JH, Dowle C, Williams MR, Elston CW, Ellis IO, Hinton CP, Blamey RW and Haybittle JL (1987) Confirmation of a prognostic index in primary breast cancer. *Br J Cancer* **56**: 489–492

Travis A, Pinder SE, Robertson JFR, Bell JA, Wencyk P, Gullick WJ, Nicholson RI, Poller DN, Blarney RW, Elston CW and Ellis IO (1996) C-erbB3 in human breast carcinoma: expression and relation to prognosis and established prognostic indicators. *British Journal of Cancer* **74**: 229–233

Vecchi M and Carpenter G (1997) Constitutive proteolysis of the ErbB-4 receptor tyrosine kinase by a unique, sequential mechanism. *J Cell Biol* **139**: 995–1003

Vecchi M, Baulida J and Carpenter G (1996) Selective cleavage of the heregulin receptor ErbB-4 by protein kinase C activation. *J Biol Chem* **271**: 18989–18995

Wen D, Suggs SV, Karunagaran D, Liu N, Cupples RL, Luo Y, Janssen AM, Ben-Baruch N, Trollinger DB and Jacobsen VL (1994) Structural and functional aspects of the multiplicity of neu differentiation factors. *Mol Cell Biol* **14**: 1909–1919

© 2000 Cancer Research Campaign

Zhang D, Sliwkowski MX, Mark M, Frantz G, Akita R, Sun Y, Hillan K, Crowley C, Brush J and Godowski PJ (1997) Neuregulin 3 (NRG3): a novel neural tissue-enriched protein that binds and activates ErbB4. *Proc Natl Acad Sci USA* **94**: 9562–9567
Zhu X, Lai C, Thomas S and Burden SJ (1995) Neuregulin receptors, erbB3 and erbB4 are localized at neuromuscular synapses. *EMBO J* **14**: 5842–5848
Zimonjic DB, Alimandi M, Miki T, Popescu NC and Kraus MH (1995) Localization of the human HER4/erbB-4 gene to chromosome 2. *Oncogene* **10**: 1235–1237

*© 2000 Cancer Research Campaign*
